# Single-case report: dynamic changes in cardiac function during shamanic journeying and Qigong meditation

**DOI:** 10.3389/fpsyg.2025.1608442

**Published:** 2025-10-31

**Authors:** Emma R. Huels, Lily Carter, Gang Xu, Jimo Borjigin, Richard E. Harris

**Affiliations:** ^1^Department of Anesthesiology, University of Michigan, Ann Arbor, MI, United States; ^2^Center for Consciousness Science, University of Michigan, Ann Arbor, MI, United States; ^3^Department of Molecular and Integrative Physiology, University of Michigan, Ann Arbor, MI, United States; ^4^Frankel Cardiovascular Center, University of Michigan, Ann Arbor, MI, United States; ^5^Neuroscience Graduate Program, University of Michigan, Ann Arbor, MI, United States; ^6^Chronic Pain and Fatigue Research Center, Department of Anesthesiology, University of Michigan, Ann Arbor, MI, United States; ^7^Susan Samueli Integrative Health Institute, School of Medicine, University of California at Irvine, Irvine, CA, United States; ^8^Department of Anesthesiology and Perioperative Care, School of Medicine, University of California at Irvine, Irvine, CA, United States

**Keywords:** heart rate variability, shamanism, Qigong, meditation, electrocardiogram, case report

## Abstract

**Introduction:**

Previous studies have demonstrated acute changes in brain activity that occur during shamanic journeying—an ancient spiritual practice used for physical, psychological, and spiritual healing. However, the effect of shamanic journeying on other physiological measures, such as heart rate variability, remains unknown. We investigated changes in heart rate variability (HRV) during sessions of shamanic journeying (*n* = 14) in a single subject, as well as during Qigong meditation (*n* = 8) in the same individual as a positive control.

**Methods:**

Electrocardiogram (ECG) signals were recorded and compared between various events during the shamanic journeys (e.g., rest, drumming, shapeshift, post-shapeshift), as well as during periods of rest and Qigong meditation. ECG signals were first visualized using the Electrocardiomatrix (ECM) software and further quantified using the following HRV measures: beats per minute (BPM), average RR interval (AVRR), standard deviation of RR interval (SDRR), root mean square of successive RR interval differences (RMSSD), and the percentage of successive RR intervals that differ by more than 50 milliseconds (pNN50). Segments > 65 s were evaluated for frequency measures, including the absolute power of the low (LF: 0.04–0.15 Hz) and high (HF: 0.15–0.4 Hz) frequency bands and the ratio of LF to HF power (LF/HF ratio).

**Results:**

The start of shamanic drumming decreased BPM and increased AVRR (compared to rest), as well as increased SDRR, RMSSD, pNN50, LF power, and LF/HF ratio. Upon shapeshifting, BPM, SDRR, and RMSSD all increased and AVRR decreased. BPM remained elevated (and AVRR remained decreased) during the first post-shapeshift period, while the LF/HF ratio decreased. The LF/HF ratio decreased further in the second post-shapeshift period, while HF power and pNN50 were increased compared to drumming initiation. Qigong meditation also increased SDRR, RMSSD, pNN50, LF power, HF power, and the LF/HF ratio compared to rest. Changes in SDRR, LF power, and LF/HF ratio during Qigong meditation were greater than those induced by drumming initiation, while changes in BPM and AVRR were greater during drumming initiation.

**Discussion:**

These data suggest that shamanic journeying involves dynamic, widespread changes in cardiac function and physiology that can be tracked visually in the ECM and captured by ultra-short-term HRV.

## 1 Introduction

Shamanic journeying is an ancient spiritual practice used for physical, psychological, and spiritual healing that involves dynamic changes in consciousness, including feelings of unity, spiritual experiences, and out of body sensations (Eliade, 1964; Harner, 1980). During journeying, a shaman (i.e., a healer within an indigenous tribe or culture) or shamanic practitioner (i.e., a healer practicing neo-shamanism in the western world) may use information acquired via interactions with the spirit world, such as power animals or deceased spiritual leaders, to help heal others or themselves (Eliade, 1964; Harner, 1980). Previous studies have demonstrated changes in brain activity during the shamanic state of consciousness induced by drumming, a common journeying technique (Hove et al., 2016; Huels et al., 2021). For example, Huels et al. (2021) measured changes in brain activity in shamanic practitioners during drumming using electroencephalography (EEG) and observed increased spectral power and decreased signal diversity in the gamma band, as well as increased metastability in the beta band. Moreover, Hove et al. (2016) found increased centrality of and coactivation between the posterior cingulate cortex, dorsal anterior cingulate cortex, and the insula using fMRI. In contrast, studies investigating changes in other physiological measures, such as heart rate variability (HRV), during shamanic journeying are lacking. As many traditions cite the heart as playing a key role in the shamanic journey (Harner, 1980), cardiac function may also vary during this practice. Additionally, while previous studies have evaluated the effects of various styles of meditation (Sun and Yan, 1992; Murata et al., 2004; Takahashi et al., 2005; Phongsuphap et al., 2008; Wu and Lo, 2008; An et al., 2010; Nesvold et al., 2012; Amihai and Kozhevnikov, 2014; Natarajan, 2023) and yoga (Muralikrishnan et al., 2012; Tyagi and Cohen, 2016) on HRV, to our knowledge, no studies have compared changes in heart rate during shamanic journeying to those seen during meditative practices such as Qigong meditation—a moving meditation that focuses on the flow of Qi, or vital energy, through controlled breathing and gentle movements (Reid, 1998). Thus, we recorded and analyzed electrocardiogram (ECG) signals from a single subject experienced in both shamanic journeying and Qigong meditation during 14 individual shamanic journeys and 8 separate meditation sessions using the Electrocardiomatrix (ECM) Mobile Device—a proprietary prototype developed in the Borjigin laboratory at the University of Michigan (Li et al., 2015). We hypothesized that shamanic journeying would result in changes in ECG signals that may also differ from Qigong meditation.

## 2 Methods

### 2.1 Participant

Informed consent was obtained from the subject prior to study procedures. All data were collected from this subject (53-year-old Caucasian American male) who had 15 years of experience in Core Shamanism with continued practice monthly and 30 years of experience in Qigong meditation with daily practice. The shamanic practice of the subject regularly consisted of passively listening to rhythmic drumming, as is common in Core Shamanism (Harner, 1980). Developed by Michael Harner, Core Shamanism, like other shamanic traditions, involves awareness of and travel between spiritual realms (Upper, Middle, and Lower Worlds) as well as shapeshifting into power animals (Harner, 1980) (see Eliade, 1964 for more information on shamanic practice). The individual’s Qigong meditation practice involved simple repeated arm motion aligned with the breath (see Cohen, 1997 and Yang, 2022 for more information regarding Qigong meditation). Of note, the subject did not have any major untreated physical or psychological conditions.

### 2.2 Data acquisition

Electrocardiogram (ECG) signals were recorded over 6 months using the Movesense MD (Movesense Ltd., Vantaa, Finland) during 14 shamanic journeys induced via rhythmic drumming and 8 Qigong meditation sessions. The ECG signals, captured by the Movesense MD sensor, were transmitted via Bluetooth to an android mobile device and converted automatically in real-time to ECM display using the Electrocardiomatrix Mobile Device software (Li et al., 2015) installed on the tablet. All experiments took place between December 2022 and July 2023 in a dimly lit room within the subject’s home between 9 PM-12 AM while the subject was either seated (Qigong) or in a supine (shamanic journeying) position. The subject received prior training in the use of the Movesense MD sensor and ECM software, enabling independent completion of all experimental procedures without experimenter intervention.

Shamanic journeys consisted of a baseline rest period, after which a 15-min or 25-min shamanic drumming recording was started. This recording consisted of rhythmic drumming (25-min) (Ingerman, 2010b) or rhythmic drumming combined with rattles (15-min) (Ingerman, 2010a). During this time, the subject would enter a non-ordinary reality state (i.e., the shamanic state of consciousness) and experience a different spiritual plane (e.g., Lower World). This was almost always (13/14 journeys) accompanied by a shapeshift period, during which the subject would either reunite with or transform into their power animal while in this altered state. Given that an individual can have multiple power animals (e.g., bear, wolf, owl, etc.), we only analyzed data from shamanic journeys that included shapeshifting into the same power animal (12/14 journeys). Additionally, since the relationship between an individual and their power animal is sacred and private, the identity of the subject’s power animal has been withheld. Both breathing and movement varied during shamanic journeys, with the greatest variations occurring during the shapeshift period, which was evident on the ECM display. During this time, movements of the subject often simulated the movements of the power animal (e.g., moving arms as if they had wings). To control for this, we recorded ECG signals during sham movement sessions where the subject moved their arms with the same range of motion as to what happened during shapeshift periods without entering the shamanic state of consciousness. Of note, the subject did not perform any intentional changes in breathing during the sham movement sessions. Qigong meditation sessions consisted of a baseline rest period, Qigong meditation, and ended with another rest period. During Qigong meditation, the subject slowly moved their arms inwards and outwards, which aligned with the subject’s inhalation and exhalation, respectively. The subject recorded written notes detailing transitions between states (e.g., start and stop of drumming or meditation) and within states (e.g., shapeshift into power animal) after all experiments.

### 2.3 Identification of analysis segments

**Figure 1:**
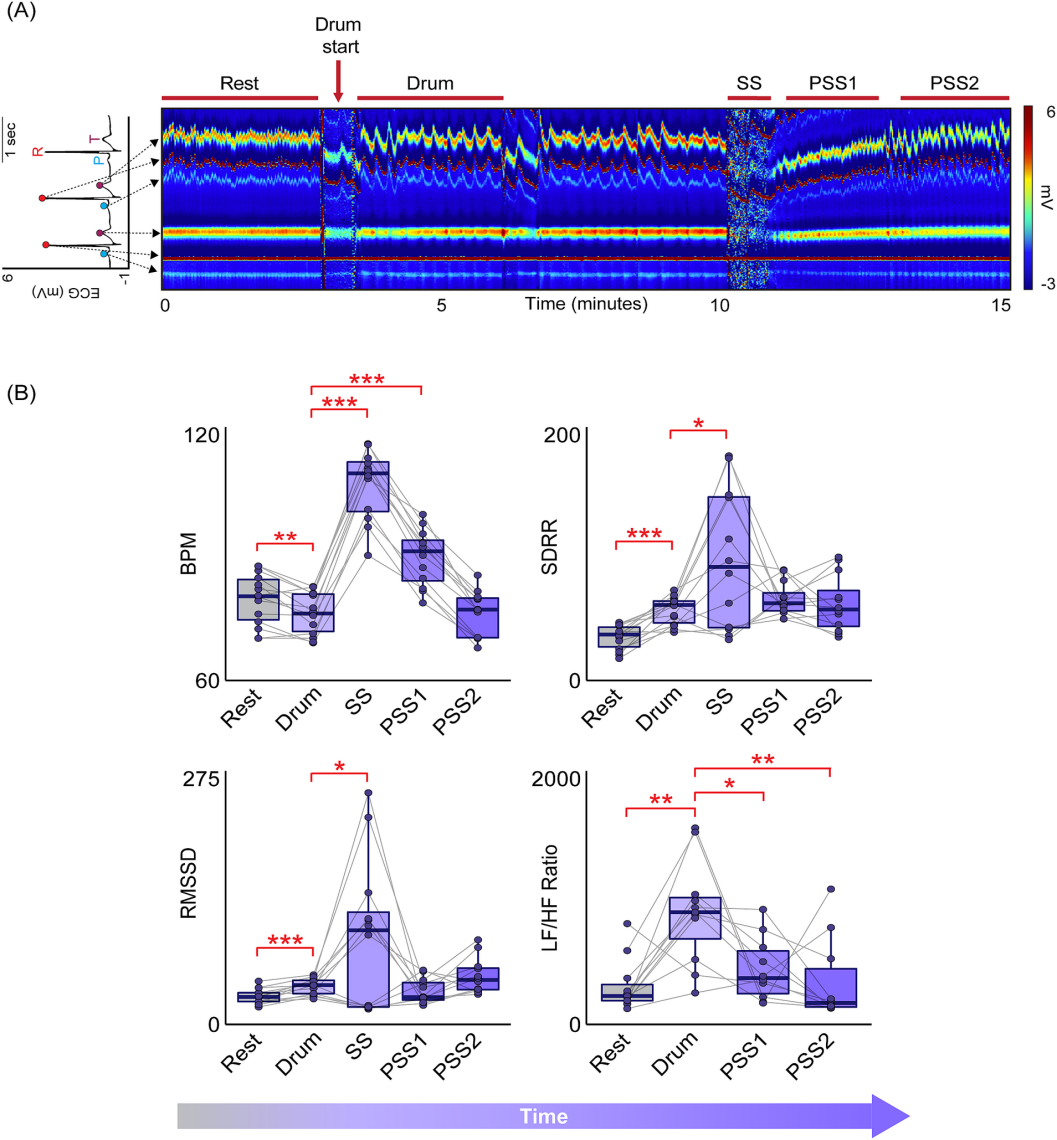
Dynamic changes in heart rate variability occur throughout the shamanic journey. **(A)** Example heat map generated by the Electrocardiomatrix (ECM) software illustrating changes in the electrocardiogram (ECG)—plotted as two successive beats—throughout a shamanic journey. Warmer colors indicate greater voltage and arrows extending from an example ECG trace indicate the location of the P, R, and T wave on the ECM heat map. **(B)** Box plots illustrating heart rate variability (HRV) measures during rest (grey) and different parts of the shamanic journey (purple). Following the start of rhythmic drumming (Drum), beats per minute (BPM) decreased while the standard deviation of the RR interval (SDRR), root mean square of successive RR interval differences (RMSSD), and the ratio of low frequency (0.04–0.15 Hz) to high frequency (0.15–0.4 Hz) power (LF/HF ratio) increased. During the shapeshift period (SS), BPM, SDRR, and RMSSD were increased compared to drumming initiation. BPM remained elevated during the first post-shapeshift period (PSS1) while the LF/HF ratio decreased (compared to the initial drumming period). The LF/HF ratio decreased further during the second post-shapeshift period (PSS2). Results for the remaining HRV measures are reported in the text. Statistical significance is indicated as follows: *p < 0.05; **p < 0.01; ***p < 0.001. Of note, the LF/HF ratio was not assessed during the shapeshift period due to the shortened (<65 s) duration.

The ECM, which illustrates changes in the electrocardiogram over time via a heat map, of each shamanic journey was visually inspected for 30–120 s of continuous artifact-free data for the following events, which were verified by the subject’s written notes: rest, drumming initiation (beginning at “drum start” in Figure 1A), shapeshift into power animal, post-shapeshift 1, and post-shapeshift 2. Post-shapeshift 1 and 2 were defined as the first and second steady-state periods visualized in the ECM immediately following the shapeshift period. These events (e.g., drumming initiation, shapeshift, post-shapeshift 1 and 2) generally took place within the first 10 min of each shamanic journey. Given the dynamic nature of each shamanic journey, we did not analyze events beyond post-shapeshift 2 in any of the journeys, as the subsequent events that took place tended to be much more variable (in content and ECM visualization) and were not easily comparable across experiments.

The duration of analysis segments was largely driven by the duration of each state within the shamanic journey, such that 120 s was the upper boundary across states (e.g., rest, drumming initiation, shapeshift, post-shapeshift 1 and 2), excluding the shapeshift period which always lasted under 1 min (mean ± SD: 42.9 s ± 8.8 s). The shorter durations (≤ 120 s) found within this study are largely due to the dynamic nature of the shamanic journey itself, as well as the unpredictability of states within each shamanic journey. For example, the drumming initiation period may last 6 min within one experiment, but only 1 min within a different experiment, simply because the subject shapeshifted sooner in the latter experiment. Moreover, the analysis segments for post-shapeshift 1 and post-shapeshift 2 were consistently 60–100 s. Thus, we restricted our analysis durations to 30–120 s to maintain consistency across states. Of note, only one rest period (30 s) and one drumming initiation period (51 s) were less than 1 min due to the necessity to select data around artifacts and rapid shapeshifting after the drumming music began, respectively. The remaining analysis segments for all states, excluding the shapeshift period, were at least 60 s (rest: 79.9 s ± 22.5 s; drumming initiation: 76.9 s ± 16.7 s; post-shapeshift 1: 77.2 s ± 13.4 s; post-shapeshift 2: 86.6 s ± 16 s). These durations are within the range proposed for studies utilizing ultra-short-term HRV (Shaffer et al., 2016; Shaffer and Ginsberg, 2017).

The Qigong meditation sessions were evaluated for continuous 120-s periods of rest (prior to meditation) and continuous 120-s periods of meditation using the subject’s notes and visual inspection of the ECM. Of note, 120 s was chosen as the duration of analysis periods for the meditation experiments to match the max duration for the shamanic journeying experiments.

### 2.4 Heart rate variability measures

HRV measurements were calculated during each state (e.g., rest, drumming initiation, etc.) for each experiment using the ECM software, which outputs temporal and frequency HRV measures for a given analysis segment. All segments were analyzed using the ECM software for the following temporal measures: the average beats per minute (BPM); the average interbeat (RR) interval (i.e., time between all heartbeats, including irregular or abnormal beats; AVRR), which is inversely related to BPM; the standard deviation of interbeat intervals (SDRR) of all sinus beats, including false or abnormal beats; the root mean square of successive interbeat interval differences (RMSSD), which is correlated with parasympathetic nervous system (PNS) activity; and the percentage of successive interbeat intervals that differ by more than 50 milliseconds (pNN50), which is also correlated with PNS activity (and in turn RMSSD). Segments longer than 65 s were evaluated for frequency measures, including the absolute power of the low (LF: 0.04–0.15 Hz) and high (HF: 0.15–0.4 Hz) frequency bands and the ratio of LF to HF power (LF/HF ratio). LF power may be influenced by sympathetic nervous system (SNS) or PNS activity (Shaffer and Ginsberg, 2017). In contrast, HF power is associated with PNS activity and is correlated with the measures RMSSD and pNN50. Although the LF/HF ratio has historically been thought to reflect sympathetic/parasympathetic balance, this belief has been challenged (Billman, 2013; Shaffer et al., 2014). For a detailed review of individual HRV measures and their meaning, please see Shaffer and Ginsberg (2017). Given the mean duration of shapeshift periods was 43 s, shapeshift segments were only evaluated for temporal and not frequency measures. Sham movement sessions were identified and analyzed in the same fashion as the shapeshift period.

### 2.5 Statistical comparisons

Paired t-tests were used to compare HRV measures between states within each experiment. For the shamanic journeying experiments, comparisons were made between (1) drumming initiation and rest, (2) shapeshift and drumming initiation, (3) post-shapeshift 1 and drumming initiation, (4) post-shapeshift 2 and drumming initiation. Given that the shapeshift period was accompanied by motion, particularly of the arms, we also compared the change in HRV measures following shapeshift (shapeshift—drumming initiation) to the change in HRV measures following simulated movements outside of the shamanic journey (motion—rest). For the control experiments conducted during Qigong meditation, HRV measures were only compared between Qigong and rest using a paired t-test. Additionally, we compared the change in HRV measures following Qigong meditation (Qigong—rest) to the change induced by shamanic drumming (drumming initiation—rest) to see how HRV during Qigong meditation and shamanic journeying differed. All statistical comparisons were made via the t.test function using R Software (RStudio version 1.1.463). We did not correct for multiple comparisons given our low sample size and the nature of this study (i.e., a case report). For each statistical comparison, Table 1 contains t-statistics, *p* values, and 95% confidence intervals, while Supplementary Table 1 provides the means and standard deviations across recording sessions for each state.

**Table 1:** Statistical comparisons during and between shamanic journeying and Qigong meditation.

HRV measure	Qigong vs. Rest				Drum vs. Rest				Qigong vs. Drum			
	*t*	*p*	95% CI	∆	*t*	*p*	95% CI	∆	*t*	*p*	95% CI	∆
BPM	−0.64	0.54	[−1.85, 1.06]	**–**	**−3.64**	**< 0.01**	**[−5.89, −1.50]**	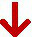	**2.78**	**0.012**	[0.82, 5.78]	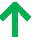
AVRR	0.53	0.61	[−9.59, 15.08]	**–**	**3.52**	**< 0.01**	**[14.54, 60.66]**	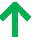	−2.93	**< 0.01**	[−59.81, −9.89]	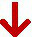
SDRR	**9.74**	**< 0.0001**	**[26.64, 43.72]**	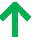	**6.76**	**< 0.0001**	**[15.95, 30.94]**	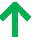	**2.34**	**0.031**	**[1.21, 22.27]**	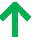
RMSSD	**3.64**	**< 0.01**	**[3.31, 15.59]**	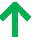	**5.28**	**< 0.001**	**[6.94, 16.55]**	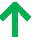	−0.67	0.51	[−9.53, 4.94]	-
pNN50	**6.69**	**< 0.001**	**[1.66, 3.47]**	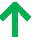	**3.96**	**< 0.01**	**[2.34, 7.93]**	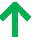	−1.90	0.076	[−5.44, 0.31]	-
LF power	**7.77**	**< 0.001**	**[456.61, 856.28]**	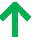	**5.45**	**< 0.001**	**[198.08, 471.80]**	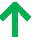	**3.08**	**< 0.01**	**[96.93, 546.08]**	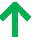
HF power	**2.82**	**0.026**	**[3.56, 40.85]**	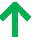	1.30	0.22	[−16.71, 63.12]	–	−0.051	0.96	[−43.12, 41.12]	–
LF/HF ratio	**4.41**	**< 0.01**	**[920.95, 3049.17]**	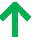	**4.15**	**< 0.01**	**[295.82, 980.01]**	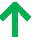	**2.83**	**0.020**	**[264.71, 2429.59]**	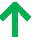

HRV measure	Shapeshift vs. Drum				Post-Shapeshift 1 vs. Drum				Post-Shapeshift 2 vs. Drum			
	*t*	*p*	95% CI	∆	*t*	*p*	95% CI	∆	*t*	*p*	95% CI	∆
BPM	**20.44**	**< 0.0001**	**[29.37, 36.46]**	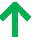	**8.07**	**< 0.0001**	**[10.14, 17.74]**	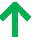	−0.55	0.59	[−2.65, 1.59]	**–**
AVRR	**−24.75**	**< 0.0001**	**[−262.59, −219.70]**	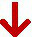	**−8.44**	**< 0.0001**	**[−154.38, −90.49]**	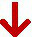	0.68	0.51	[−15.42, 29.23]	**–**
SDRR	**2.97**	**0.013**	**[11.80, 79.16]**	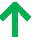	2.20	0.050	[−0.016, 22.29]	–	1.07	0.31	[−7.54, 21.91]	**–**
RMSSD	**2.53**	**0.028**	**[7.90, 113.39]**	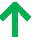	−0.90	0.39	[−15.93, 6.67]	–	2.17	0.052	[−0.17, 26.83]	**–**
pNN50	−0.22	0.83	[−6.93, 5.68]	-	−0.24	0.81	[−6.96, 5.58]	–	**2.62**	**0.024**	**[1.68, 19.29]**	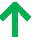
LF power	–	–	-	–	0.34	0.74	[−287.72, 389.77]	–	0.10	0.92	[−356.31, 390.92]	-
HF power	–	–	–	–	1.71	0.12	[−18.08, 130.37]	–	**3.24**	**0.010**	**[48.12, 270.55]**	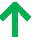
LF/HF ratio	–	–	–	–	**−2.79**	**0.021**	**[−899.92, −93.76]**	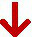	**−4.15**	**< 0.01**	**[−927.64, −273.14]**	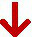

Paired t-tests were used to make the following statistical comparisons for each heart rate variability (HRV) measure: Qigong vs. Rest; shamanic drumming initiation (Drum) vs. Rest; Shapeshift, Post-Shapeshift 1 or Post-Shapeshift 2 vs. Drum; Qigong-Rest vs. Drum-Rest. The corresponding *t* value, *p* value, and lower/upper 95% confidence interval are listed for each statistical comparison. Bold values represent *p* < 0.05. Arrows indicate the direction of change for each HRV measure during the first state listed for each comparison (e.g., for Drum vs. Rest, BPM was decreased during Drum). BPM, beats per minute; AVRR, average RR interval; SDRR, standard deviation of RR interval; RMSSD, root mean square of successive RR interval differences; pNN50, percentage of successive RR intervals that differ by more than 50 milliseconds; LF power, low frequency power; HF power, high frequency power; LF/HF ratio, ratio of LF to HF power; *t*, test statistic; *p*, significance; CI, confidence interval [lower, upper]; 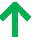, increase in HRV measure; 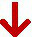, decrease in HRV measure.

## 3 Results

### 3.1 Dynamic changes in HRV measures occur throughout the shamanic journey

**Figure 2:**
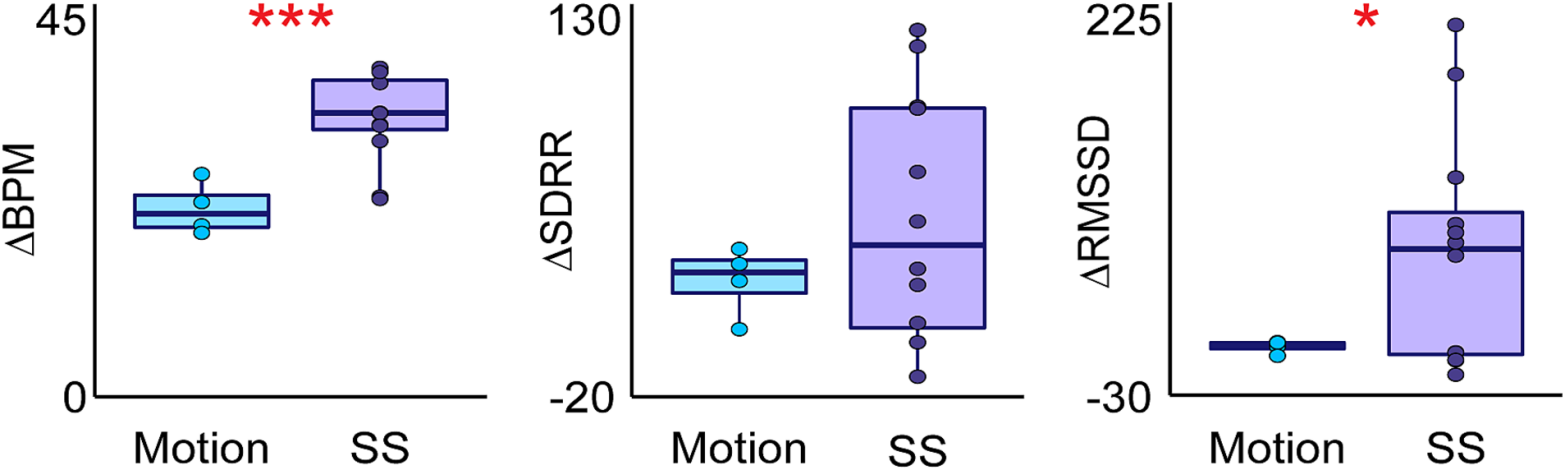
Differences in heart rate variability measures during shamanic journeying and sham movement sessions. Box plots comparing the change in heart rate variability (HRV) measures during sham motion (Motion—Rest; blue) and the shapeshift (Shapeshift (SS)—Rest; purple) periods. The change in beats per minute (BPM) and the root mean square of successive RR interval differences (RMSSD) was greater during the SS period compared to Motion, suggesting that these changes are not simply due to movement. Statistical significance is indicated as follows: *p < 0.05; ***p < 0.001.

An example heat map generated by the ECM software for a single shamanic journey is illustrated in Figure 1A. Following the start of shamanic drumming, there was a decrease in BPM (*t*(13) = −3.64, *p* < 0.01) and increase in AVRR (*t*(13) = 3.52, *p* < 0.01), SDRR (*t*(13) = 6.76, *p* < 0.0001), RMSSD (*t*(13) = 5.28, *p* < 0.001), pNN50 (*t*(13) = 3.96, *p* < 0.01), LF power (*t*(10) = 5.45, *p* < 0.001), and LF/HF ratio (*t*(10) = 4.15, *p* < 0.01) compared to rest. Upon shapeshifting, AVRR decreased (*t*(10) = −24.75, *p* < 0.0001) and BPM increased (*t*(11) = 20.44, *p* < 0.0001) compared to drumming initiation, as did SDRR (*t*(11) = 2.97, *p* = 0.013) and RMSSD (*t*(11) = 2.53, *p* = 0.028). The changes in BPM (*t*(11) = 8.067, *p* < 0.0001) and AVRR (*t*(11) = −8.44, *p* < 0.0001) extended into the post-shapeshift 1 period and the LF/HF ratio became decreased compared to drumming initiation (*t*(9) = −2.79, *p* = 0.021). Compared to the initial drumming period, the LF/HF ratio decreased even further during post-shapeshift 2 (*t*(9) = −4.15, *p* < 0.01), while HF power (*t*(9) = 3.24, *p* = 0.01) and pNN50 (*t*(11) = 2.62, *p* = 0.024) increased (Figure 1B). Of note, changes in BPM (*t*(9.05) = 4.91, *p* < 0.001), AVRR (*t*(13.67) = −6.14, *p* < 0.0001), and RMSSD (*t*(11.19) = 2.80, *p* = 0.017) were greater following shapeshift as compared to following simulated movements (Figure 2).

### 3.2 Qigong meditation leads to widespread changes in HRV measures

**Figure 3:**
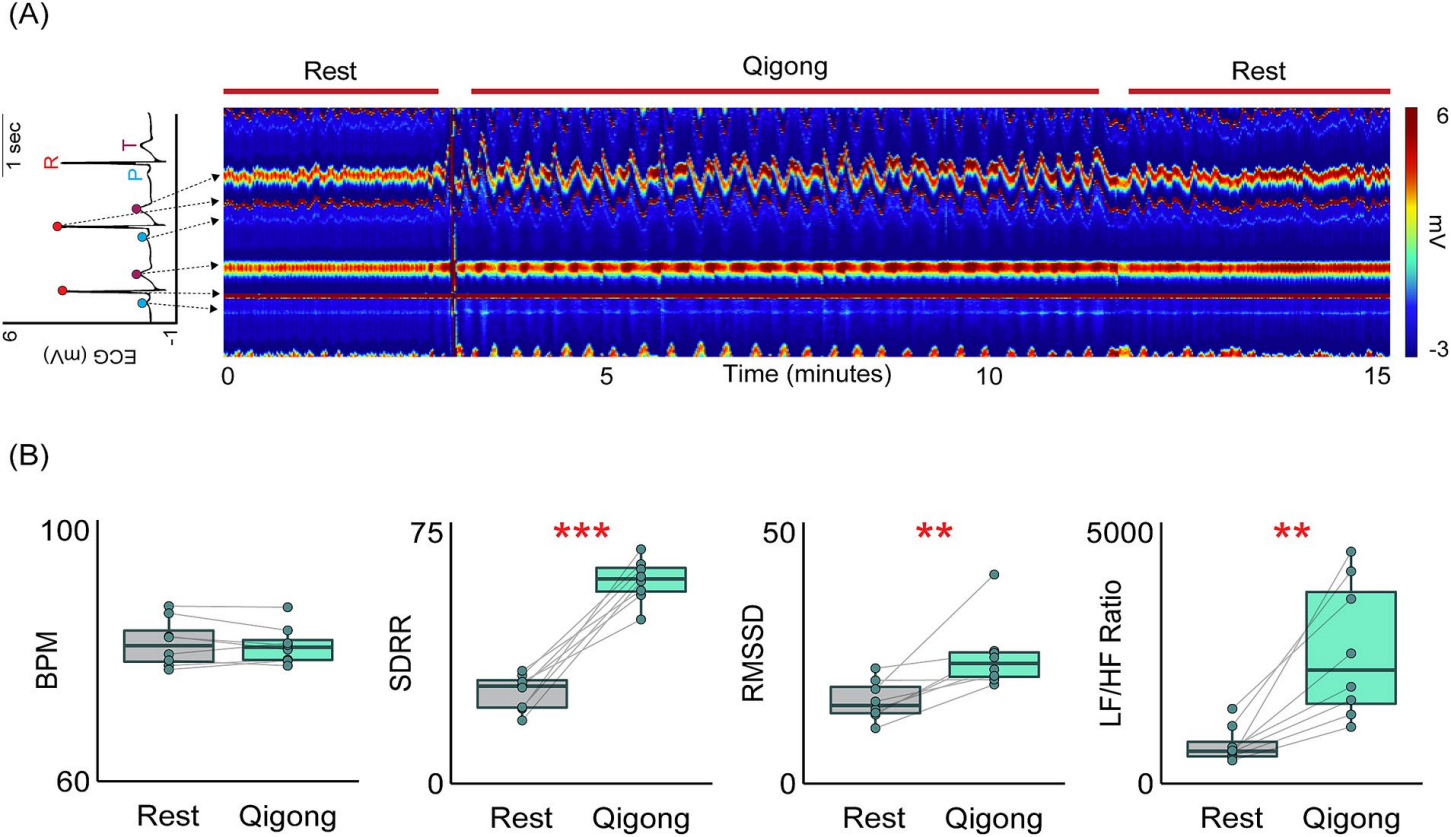
Heart rate variability increases during Qigong meditation. **(A)** Example heat map generated by the Electrocardiomatrix (ECM) software illustrating changes in the electrocardiogram (ECG)—plotted as two successive beats—during periods of rest and Qigong meditation. Warmer colors indicate greater voltage and arrows extending from an example ECG trace indicate the location of the P, R, and T wave on the ECM heat map. **(B)** Box plots showing heart rate variability (HRV) measures during rest (grey) and Qigong meditation (green). Qigong meditation increased the standard deviation of the RR interval (SDRR), root mean square of successive RR interval differences (RMSSD), and the ratio of low frequency (0.04–0.15 Hz) to high frequency (0.15–0.4 Hz) power (LF/HF ratio) while beats per minute (BPM) remained unchanged. Results for the remaining HRV measures are reported in the text. Statistical significance is indicated as follows: **p < 0.01; ***p < 0.001.

An example heat map generated by the ECM software for a single Qigong meditation session is illustrated in Figure 3A. Compared to rest, Qigong meditation led to an increase in SDRR (*t*(7) = 9.74, *p* < 0.0001), RMSSD (*t*(7) = 3.64, *p* < 0.01), pNN50 (*t*(7) = 6.69, *p* < 0.001), LF power (*t*(7) = 7.77, *p* < 0.001), HF power (*t*(7) = 2.82, *p* = 0.026), and LF/HF ratio (*t*(7) = 4.41, *p* < 0.01). There was no change in the BPM (*t*(7) = −0.64, *p* = 0.54) or AVRR (*t*(7) = 0.53, *p* = 0.61) (Figure 3B).

### 3.3 Changes in HRV vary between Qigong meditation and shamanic journeying

**Figure 4:**
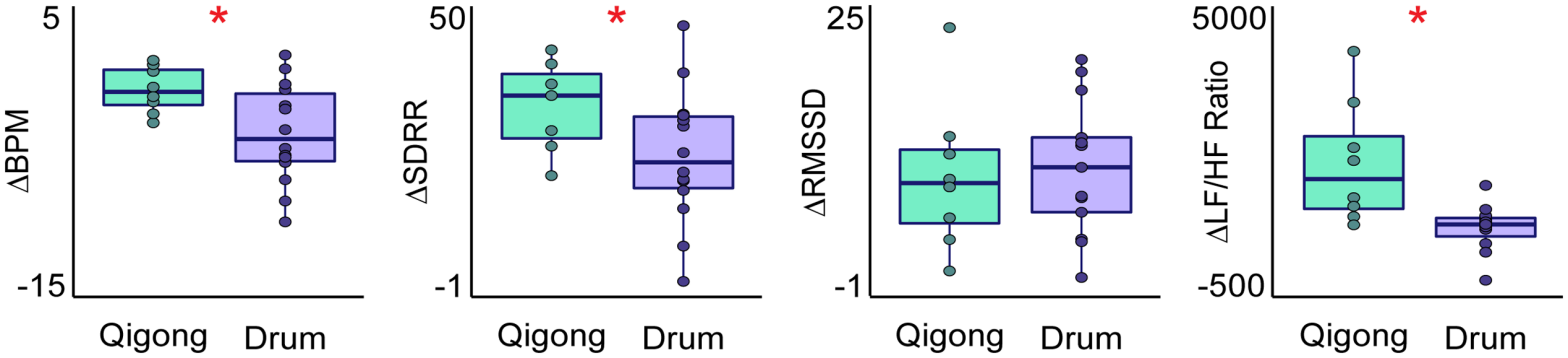
Differences in the change in heart rate variability measures during Qigong meditation and shamanic journeying. Box plots show the change in HRV measures during Qigong meditation (Qigong—Rest; green) and shamanic drumming initiation (Drum—Rest; purple). Initiation of shamanic drumming led to a greater decrease in BPM compared to Qigong meditation, which did not alter BPM. There were greater increases in the standard deviation of the RR interval (SDRR) and the ratio of low frequency (0.04–0.15 Hz) to high frequency (0.15–0.4 Hz) power (LF/HF ratio) during Qigong meditation compared to shamanic drumming initiation. Changes in RMSSD from rest to Qigong meditation or drumming initiation were similar. Statistical significance is indicated as follows: *p < 0.05.

Lastly, we compared the change in HRV measures from rest during Qigong meditation and shamanic journeying experiments (i.e., Qigong—Rest vs. Drum initiation—Rest). Shamanic drumming initiation led to decreased BPM (*t*(19.44) = 2.78, *p* = 0.012) and increased AVRR (*t*(18.04) = −2.93, *p* < 0.01) compared to Qigong meditation, during which BPM and AVRR were unchanged from rest. Increases in SDRR (*t*(17.74) = 2.34, *p* = 0.031), LF power (*t*(13.68) = 3.08, *p* < 0.01), and LF/HF ratio (*t*(8.64) = 2.83, *p* = 0.02) were greater following Qigong meditation as compared to shamanic drumming initiation. Changes in RMSSD (*t*(16.3) = −0.67, *p* = 0.51), pNN50 (*t*(15.16) = −1.9, *p* = 0.076), and HF power (*t*(13.52) = −0.051, *p* = 0.96) did not differ between the Qigong meditation and shamanic drumming initiation (Figure 4).

## 4 Discussion

To our knowledge, this is the first study to measure HRV during shamanic journeying, as well as to compare HRV across different types of spiritual altered states of consciousness. Our results demonstrate that both the initiation of shamanic drumming and Qigong meditation were characterized by an increase in HRV, with larger changes occurring during Qigong mediation. Specifically, there was an increase in SDRR, RMSSD, pNN50, LF power, and the ratio of LF/HF power during both shamanic drumming initiation and Qigong meditation. However, BPM decreased (and AVRR increased) during shamanic drumming initiation, but not during Qigong meditation whereas HF power increased during Qigong meditation (but not during shamanic drumming initiation). Therefore, while there was strong overlap between the two states, the changes engendered by shamanic drumming and Qigong meditation were distinct.

Although we observed dynamic changes in HRV during shamanic journeying, we only compared changes in HRV during the initial shamanic drumming period (i.e., prior to shapeshift) to Qigong meditation. We reasoned that these states were the most comparable. In contrast, comparisons for the shapeshift and post-shapeshift period were less straightforward, as they were more dynamic, reflecting “active” states possibly characterized by more cognitive engagement. Notably, HRV fluctuated throughout the shamanic journey with respect to distinct events, such as shapeshifting into a power animal. While future studies are needed to determine the significance of such changes, these data suggest that ultra-short-term HRV recordings may be a useful tool for tracking changes during dynamic states of consciousness such as shamanic journeying.

Given that ultra-short-term HRV is still a burgeoning field, the interpretation of the results of the current paper is somewhat difficult. Traditionally, an increase in LF/HF ratio is interpreted as a shift towards sympathetic dominance (Shaffer et al., 2014). However, given that there was an increase in both LF power and HF power, as well as an increase in RMSSD and pNN50, it is more likely that both the sympathetic and parasympathetic nervous system were activated during shamanic drumming initiation and Qigong meditation, with potentially a dominance in sympathetic activity. While frequency measures could not be measured during the shapeshift period due to the short duration of data, it is likely that this period was dominated by sympathetic activity, especially given the increased heart rate and anecdotal increase in breathing. This was followed by an increase in HF power during the post-shapeshift 2 period and decrease in the LF/HF ratio, likely reflecting a shift towards parasympathetic dominance. Overall, the balance of sympathetic and parasympathetic activity appeared to fluctuate throughout the shamanic journey.

Few studies have investigated heart rate variability during or after Qigong meditation, but previous literature often cites an increase in heart rate (or decrease in the R-R interval) during or immediately after Qigong meditation (Lin et al., 2018; Goldbeck et al., 2021). Goldbeck et al. (2021) also found an increased SDNN (similar to SDRR) during Qigong meditation, similar to our study, but RMSSD decreased whereas we observed an increase. Of note, other studies examining HRV during meditative practice have found increased LF and HF power during nondirected meditation (Nesvold et al., 2012), as well as increased HF power and decreased LF/HF ratio during Theravada meditative practices (Amihai and Kozhevnikov, 2014). In contrast, LF power, in addition to the LF/HF ratio, were found to increase during Vipassana meditation (Delgado-Pastor et al., 2013). Thus, changes in HRV during meditative practices seem to vary widely based on the practice itself, making the comparison of HRV changes we observed during shamanic journeying (or Qigong meditation) to other spiritual or meditative practices difficult. A larger sample size and more studies measuring HRV during shamanic journeying and Qigong meditation are needed to assess the differential impact of these spiritual practices on HRV. Moreover, given that HRV is nonlinear (Young and Benton, 2015), future studies should also aim to analyze changes during shamanic journeying (and meditation) using more sophisticated, nonlinear approaches.

The findings of the current study support the use of ultra-short-term HRV to measure fluctuations in heart rate during contemplative practices. In addition to investigating changes in HRV in a wider population of shamanic practitioners, future studies could also investigate changes in HRV during other spiritual practices (e.g., prayer, recitation, trance, etc.). Additionally, future studies should aim to assess whether shamanic practice leads to long-term changes in HRV in the shamanic healer or the individual being healed. Given previous literature demonstrating changes in brain activity during shamanic trance (Hove et al., 2016; Flor-Henry et al., 2017; Huels et al., 2021; Rogerson et al., 2021), these data support future investigations of changes in HRV paired with dynamic measurement of neurophysiological measures such as electroencephalography and functional magnetic resonance imaging.

This study is not without limitations. First and foremost, future studies are needed to determine the reproducibly of the findings of this single-case report in a larger population. Given the small number of subjects (*n* = 1) and experiments (*n* = 14 for shamanic journeying and *n* = 8 for Qigong meditation), the statistical results reported in this manuscript should be interpreted with caution. Future studies with a larger number of subjects and experiments could utilize non-parametric or repeated measures approaches to account for the assumptions needed to be made for parametric testing, as well as correct for multiple comparisons. Of note, at least half of the findings reported in this manuscript survive even when correcting for multiple comparisons (*p* = 0.01 when correcting for five tests – BPM/AVRR, SDRR, RMSSD/pNN50, LF power, HF power; Bonferroni correction). Additionally, it is important to note the potential influence of breathing on HRV measures within this study. We opted to have the subject conduct their practice as naturally as possible and not constrain breathing, which is an integral factor in both practices but may impact HRV measures. Another limitation is that the segments used for analysis in the current study (≤ 120 s) are short, particularly those for the shapeshift period (< 60 s), compared to other HRV studies and are considered ultra-short-term HRV measurements—a growing field of investigation (Shaffer et al., 2016; Shaffer and Ginsberg, 2017). We would argue that the length of segments utilized, particularly for shamanic journeying datasets, is warranted due to the dynamic nature of the journey itself. However, the results of this paper, particularly those from the shapeshift period, should be interpreted with caution until future studies can replicate these results. Finally, the subject annotated events of the shamanic journey following the end of the recording. While this may have impacted the temporal resolution of the time points linked to different events during the journey, this avoided interruptions to the journey itself, which was necessary for the subject to enter and maintain the shamanic state of consciousness.

In summary, we demonstrate that shamanic journeying and Qigong meditation involve dynamic, widespread changes in cardiac function and physiology, with shamanic journeying showing the greatest variation and complexity. While future studies are needed to determine the clinical significance of these results, these data provide support for the use of ultra-short-term HRV measurements to assess dynamic states of differing contemplative or spiritual practices.

## Data Availability

The raw data supporting the conclusions of this article will be made available by the authors, without undue reservation.
