# Supplementary material for: Single-case report: dynamic changes in cardiac function during shamanic journeying and Qigong meditation
Source: Front Psychol. 2025 Oct 31;16:1608442. doi: 10.3389/fpsyg.2025.1608442 (PMC12615198; doi:10.3389/fpsyg.2025.1608442)
Supplement: Supplementary file 1 [file Table_1.DOCX]

| **HRV Measure** | **Qigong** | **Rest** | **Drum^†^** | **Rest** | **∆ Qigong** | **∆ Drum^†^** |  |  |
| --- | --- | --- | --- | --- | --- | --- | --- | --- |
| BPM | 80.85 ± 3.21 | 81.25 ± 4.01 | 74.46 ± 5.32 | 78.16 ± 6.65 | -0.40 ± 1.74 | -3.69 ± 3.80 |  |  |
| AVRR | 742.61 ± 28.12 | 739.86 ± 35.59 | 809.32 ± 59.12 | 771.72 ± 66.51 | 2.75 ± 14.75 | 37.60 ± 39.94 |  |  |
| SDRR | 60.30 ± 6.77 | 25.12 ± 5.61 | 51.49 ± 11.86 | 28.05 ± 10.49 | 35.18 ± 10.21 | 23.45 ± 12.97 |  |  |
| RMSSD | 24.44 ± 7.39 | 14.99 ± 4.19 | 31.45 ± 9.37 | 19.7 ± 8.99 | 9.45 ± 7.35 | 11.75 ± 8.32 |  |  |
| pNN50 | 2.89 ± 0.90 | 0.33 ± 0.70 | 9.21 ± 6.92 | 4.08 ± 6.58 | 2.57 ± 1.08 | 5.13 ± 4.84 |  |  |
| LF power | 736.01 ± 228.28 | 79.57 ± 58.36 | 408.86 ± 188.36 | 73.92 ± 56.39 | 656.45 ± 239.03 | 334.94 ± 203.72 |  |  |
| HF power | 40.56 ± 27.68 | 18.36 ± 10.43 | 69.21 ± 75.57 | 46.01 ± 38.80 | 22.20 ± 22.30 | 23.20 ± 59.41 |  |  |
| LF/HF ratio | 2545.17 ± 1432.84 | 560.11 ± 378.01 | 874.08 ± 448.83 | 236.17 ± 225.87 | 1985.06 ± 1272.83 | 637.91 ± 509.21 |  |  |
|  | | | | |  |  |  |  |
| **HRV Measure** | **Drum^§^** | **Shapeshift** | **Post-Shapeshift 1** | **Post-Shapeshift 2** |  |  |  |  |
| BPM | 75.13 ± 5.28 | 108.05 ± 9.15 | 89.07 ± 7.37 | 74.60 ± 5.92 |  |  |  |  |
| AVRR | 801.04 ± 57.30 | 559.89 ± 49.31 | 678.60 ± 56.16 | 807.94 ± 66.41 |  |  |  |  |
| SDRR | 50.035 ± 12.24 | 95.52 ± 60.34 | 61.17 ± 14.84 | 57.22 ± 24.43 |  |  |  |  |
| RMSSD | 30.53 ± 9.73 | 91.18 ± 90.001 | 25.90 ± 14.82 | 43.86 ± 21.16 |  |  |  |  |
| pNN50 | 8.78 ± 7.40 | 8.16 ± 11.01 | 8.09 ± 9.77 | 19.27 ± 14.94 |  |  |  |  |
| LF power | 410.71 ± 198.44 | - | 461.73 ± 538.51 | 428.01 ± 534.22 |  |  |  |  |
| HF power | 71.39 ± 79.30 | - | 127.53 ± 99.90 | 230.72 ± 200.46 |  |  |  |  |
| LF/HF ratio | 879.14 ± 472.77 | - | 382.30 ± 275.23 | 278.75 ± 364.31 |  |  |  |  |
| Mean +/- Standard Deviation; Drum^†^ and Drum**^§^** are the series of files used for a given statistical comparison (e.g., Drum^†^ vs Rest, etc.) | | | | | | | |  |

**Supplemental Table 1: Mean and Standard Deviation for HRV Measures for Each Statistical Comparison.**

|  |  |
| --- | --- |
|  |  |
|  |  |
|  |  |
|  |  |
|  |  |
|  |  |
|  |  |
|  |  |
